# Random mutagenesis screen shows that *Phytophthora capsici* CRN83_152‐mediated cell death is not required for its virulence function(s)

**DOI:** 10.1111/mpp.12590

**Published:** 2017-10-24

**Authors:** Tiago M. M. M. Amaro, Gaëtan J. A. Thilliez, Rory A. Mcleod, Edgar Huitema

**Affiliations:** ^1^ Division of Plant Sciences, School of Life Sciences University of Dundee at the James Hutton Institute (JHI), Invergowrie Dundee DD2 5DA UK; ^2^ Dundee Effector Consortium, JHI, Invergowrie Dundee DD2 5DA UK; ^3^ Cell and Molecular Sciences JHI, Invergowrie Dundee DD2 5DA UK

**Keywords:** cell death, crinkler (CRN), effector, nucleus, *Phytophthora capsici*, virulence

## Abstract

With the increasing availability of plant pathogen genomes, secreted proteins that aid infection (effectors) have emerged as key factors that help to govern plant–microbe interactions. The conserved CRN (CRinkling and Necrosis) effector family was first described in oomycetes by their capacity to induce host cell death. Despite recent advances towards the elucidation of CRN virulence functions, the relevance of CRN‐induced cell death remains unclear. *In planta* over‐expression of PcCRN83_152, a CRN effector from *Phytophthora capsici*, causes host cell death and boosts *P. capsici* virulence. We used these features to ask whether PcCRN83_152‐induced cell death is linked to its virulence function. By randomly mutating this effector, we generated PcCRN83_152 variants with no cell death (NCD) phenotypes, which were subsequently tested for activity towards enhanced virulence. We showed that a subset of PcCRN83_152 NCD variants retained their ability to boost *P. capsici* virulence. Moreover, NCD variants were shown to have a suppressive effect on PcCRN83_152‐mediated cell death. Our work shows that PcCRN83_152‐induced cell death and virulence function can be separated. Moreover, if these findings hold true for other cell death‐inducing CRN effectors, this work, in turn, will provide a framework for studies aimed at unveiling the virulence functions of these effectors.

## Introduction

Disease epidemics form one of the biggest constraints to crop growth and yield worldwide (Fisher *et al*., 2012; Oerke, 2006). Amongst the pathogens wreaking havoc on dicot crops, the oomycetes represent one of the greatest threats to global food production (Kamoun *et al*., 2015; Lamour *et al*., 2007). Within the oomycetes, *Phytophthora* species form an extensive and diverse genus of plant pathogens that collectively affect virtually all dicot plants on earth (Kroon *et al*., 2012). For example, *P. infestans*, the causal agent of potato and tomato late blight (Fry *et al*., 2015), *P. sojae* (Tyler, 2007) and *P. capsici* (Lamour *et al*., 2012) collectively cause billions of dollars’ worth of losses on potato, tomato, soybean and pepper, respectively. In addition, and more recently, the emergence of *Phytophthora* species such as *P. ramorum* (Brasier and Webber, 2010), *P. kernoviae* (Brasier *et al*., 2005) and *P. lateralis* (Green *et al*., 2013), which affect trees and shrubs, has meant that members of this genus have also become a major threat to natural ecosystems. Given their importance, there is a critical need to understand the biology of *Phytophthora* spp., their hosts and the infection process.

Plants are continuously bombarded by a diverse array of microbes that can cause disease. In most cases, infection is limited through the perception of microbe‐ or pathogen‐associated molecular patterns (MAMPs or PAMPs) by pattern recognition receptors (PRRs). Recognition results in pattern‐triggered immunity (PTI) and features a marked shift in cellular activity towards defence, defeating the vast majority of microbes (Boller and Felix, 2009; Jones and Dangl, 2006; Muthamilarasan and Prasad, 2013; Zhang and Zhou, 2010). In a select few cases and per definition, pathogens successfully infect plants of a given species. This suggests that host immune responses are suppressed or evaded—a pathogen characteristic that suggests an evolutionary basis for specialization. Genome sequencing projects, combined with the development of computational pipelines and high‐throughput functional assays, have led to the identification of factors responsible for pathogen virulence (or pathogenicity) and therefore have revolutionized our thinking about plant pathogens. State‐of‐the‐art models emanating from functional genomics, biochemical and genetic studies describe pathogen molecules that are secreted from the pathogen and delivered into host tissues (effectors), where they subvert host immunity and trigger susceptibility (Hogenhout *et al*., 2009; Jones and Dangl, 2006; Kamoun, 2007; Win *et al*., 2012).

The availability and study of *Phytophthora* genome sequences have identified large and highly diverse candidate effector repertoires, with possible roles in the infection process (Bozkurt *et al*., 2012; Oliveira‐Garcia and Valent, 2015; Schornack *et al*., 2009). Generally, these effectors can be categorized into two major classes, defined by the host compartments in which these proteins and their respective molecular targets function. Apoplastic effectors accumulate in the host apoplast, where they target surface exposed or secreted host factors, often required or associated with defence (Doehlemann and Hemetsberger, 2013). Members of the cytoplasmic effector class, however, are thought to traverse the host cell membrane and accumulate in distinct subcellular compartments, where they act on their respective host target(s) (Hein *et al*., 2009; Kamoun, 2007). Efforts aimed at defining the effector repertoires in *Phytophthora* have led to the identification of two cytoplasmic effector subclasses, named the RXLRs and CRNs (CRinkling and Necrosis). These two effector groups are defined by the presence of relatively conserved N‐terminal domains that are thought to be involved in effector translocation processes. The RXLR N‐terminal domains contain an RXLR amino acid motif (after which this effector family is named) which is thought to be essential for RXLR translocation into plant cells (Haas *et al*., 2009; Rehmany *et al*., 2005; Whisson *et al*., 2007). In the case of CRN effectors, an N‐terminal LXLFLAK motif has been shown to be required for CRN effector translocation (Schornack *et al*., 2010). The LXLFLAX motif sits between strands 2 and 3 of a ubiquitin‐like domain which is believed to be important in CRN translocation processes, but does not appear to be present in all CRN N‐termini (Zhang *et al*., 2016). Although detailed functional studies have provided great insights into RXLR protein function (Anderson *et al*., 2015), the CRN protein family has thus far been understudied (Amaro *et al*., 2017).

CRN proteins were originally discovered in a functional genomics study in which predicted secreted proteins from *P. infestans* were cloned and expressed *in planta* (Torto *et al*., 2003). Ectopic expression of CRN1 and CRN2 led to a CRN phenotype, which was presumed to reflect (an) effector function(s) and after which these proteins were named (Torto *et al*., 2003). Subsequent studies then led to the identification of additional family members in *P. infestans* and other *Phytophthora* species initially, followed by their discovery in distantly related organisms. These results have led to the suggestion that, collectively, the CRNs form an ancient protein family, emerging before the RXLR effectors (Amaro *et al*., 2017; Haas *et al*., 2009; Stam *et al*., 2013b; Zhang *et al*., 2016).

In addition to their discovery in a number of plant pathogens, CRN proteins have been implicated as virulence factors in a variety of plant–pathogen model systems. *Phytophthora sojae* lines in which PsCRN63 and PsCRN115 expression is reduced exhibit a reduced virulence phenotype on soybean, suggestive of virulence functions (Liu *et al*., 2011). Consistent with this observation, transgenic *Arabidopsis* plants that express PsCRN63 are more susceptible to *Pseudomonas syringae* and *P. capsici* (Li *et al*., 2016) indicating immune suppression that favours pathogen infection. Over‐expression of another *P. sojae* CRN (PsCRN70) in *Nicotiana benthamiana* has also been shown to enhance the susceptibility of these plants to *Phytophthora parasitica* (Rajput *et al*., 2014). Furthermore, over‐expression of *P. sojae* PsCRN108 enhanced *Arabidopsis* and *N. benthamiana* susceptibility to *P. capsici* infections. In addition, silencing of PsCRN108 reduced *P. sojae* virulence on soybean (Song *et al*., 2015). Similarly, ectopic expression of the *P. infestans* effector PiCRN8 in *N. benthamiana* leaves led to an increase in susceptibility to *P. infestans* (van Damme *et al*., 2012). One CRN effector from *P. capsici*, PcCRN83_152 (also named PcCRN4), has also been shown to be important for virulence, as transient over‐expression of this effector in *N. benthamiana* leaves enhanced *P. capsici* growth (Mafurah *et al*., 2015; Stam *et al*., 2013b). These results were further substantiated in experiments with transgenic *P. capsici* lines in which PcCRN83_152 expression was suppressed (silencing), showing reduced growth in both *Arabidopsis* and *N. benthamiana* leaves (Mafurah *et al*., 2015). In contrast with the RXLR family, members of the CRN protein family can be found in a wide range of eukaryotic organisms (Zhang *et al*., 2016). Virulence functions for CRN effectors from outside the *Phytophthora* genus have also been demonstrated. AeCRN13, a CRN effector from the oomycete *Aphanomyces euteiches*, has been shown to enhance *P. capsici* virulence when transiently over‐expressed in *N. benthamiana* leaves (Ramirez‐Garcés *et al*., 2015).

Although CRNs are thought to aid in infection, the exact mechanisms by which these proteins function remain largely unknown (Amaro *et al*., 2017). However, there have been a small number of studies in which the virulence mechanisms of CRN effectors have been described. The *P. infestans* PiCRN8 C‐terminal domain has been reported to show similarity to plant serine/threonine kinases. Moreover, it was confirmed by biochemical assays that the C‐terminal domain of PiCRN8 showed kinase activity (van Damme *et al*., 2012). However, the connection between PiCRN8 kinase activity and its virulence function remains unclear. More detailed CRN virulence function is known for *P. sojae* PsCRN108. This CRN has been shown to enhance pathogen virulence by targeting the promotor regions of genes encoding for heat shock proteins (HSPs) and thereby reducing their expression (Song *et al*., 2015). The *P. sojae* effector PsCRN63 has also been suggested to increase pathogen virulence by direct interaction and destabilization of host catalases (Zhang *et al*., 2015a). Another study has shown that over‐expression of AeCRN13, a CRN effector from the oomycete *Aphanomyces euteiches*, enhances *N. benthamiana* susceptibility to *P. capsici* by binding host chromatin and triggering DNA damage responses (Ramirez‐Garcés *et al*., 2015). In *P. capsici*, PcCRN12–997 has been shown to bind to a tomato transcription factor, SlTCP14–2, inhibiting its association with DNA. Over‐expression of SlTCP14–2 has been shown to enhance immunity against *P. capsici*, a phenotype abolished by PcCRN12–997 over‐expression (Stam *et al*., 2013c).

Although CRN functions have started to emerge, the biological relevance of cell death induced by some, but not all, CRN proteins is yet to be resolved. Several CRNs have been shown to work as suppressors of cell death and host defence responses (Rajput *et al*., 2014; Shen *et al*., 2013). Intriguingly, CRNs with high sequence similarity can also show opposite effects on cell death‐inducing activity, as is the case for PsCRN63 and PsCRN115 (Liu *et al*., 2011), and PsCRN171–1 and PsCRN171–2 (Shen *et al*., 2013). Although no virulence functions have been described for PsCRN171–1 and PsCRN171–2, studies on PsCRN63 and PsCRN115 have shown that the cell death‐inducing PsCRN63 enhances plant susceptibility, whereas the cell death‐suppressing PsCRN115 has the opposite effect (Zhang *et al*., 2015a, 2015b), suggesting a link between virulence function and CRN‐mediated cell death. However, PsCRN115 has been shown to be a potent suppressor of PsCRN63‐mediated cell death whilst having no effect on PsCRN63 virulence boost, suggesting that PsCRN63‐mediated cell death and virulence boost consist of two independent mechanisms (Zhang *et al*., 2015a). Further insights into the biological meaning of CRN‐mediated cell death were obtained in a recent work analysing the cell death‐inducing capacities of a CRN effector (PhCRN37) from *Plasmopara halstedii* in sunflower near‐isogenic lines (NILs) (Gascuel *et al*., 2016). PhCRN37 was shown to induce cell death only in resistant sunflower NILs carrying the *Pl5* resistance locus and not in the corresponding sunflower susceptible parent. These observations suggest that PhCRN37‐mediated cell death is associated with recognition events putatively mediated by one of the numerous resistance gene analogues encoded in the *Pl5* locus (Gascuel *et al*., 2016). From these studies, it is apparent that the role(s) of cell death in the biology of CRN and pathogen virulence strategies has yet to be clarified.

Here, we have attempted to gain new insights into the role(s) of CRN‐mediated cell death in virulence function using PcCRN83_152 as a model. Over‐expression of PcCRN83_152 enhances *P. capsici* virulence and induces plant cell death (Mafurah *et al*., 2015; Stam *et al*., 2013a, 2013b). Moreover, this CRN has also been shown to mediate host chromatin re‐localization when over‐expressed in *N. benthamiana* leaves (Stam *et al*., 2013a, 2013b). In order to test whether PcCRN83_152‐mediated cell death is required for its virulence function(s), a polymerase chain reaction (PCR)‐based random mutagenesis screen was performed on the PcCRN83_152 C‐terminal domain (the domain responsible for both PcCRN83_152 virulence and cell death functions) (Mafurah *et al*., 2015; Stam *et al*., 2013a, 2013b). This screen allowed the identification of PcCRN83_152 variants that, despite not causing cell death, retained the capacity to enhance *P. capsici* virulence. Furthermore, no cell death (NCD) variants showed a capacity to repress PcCRN83_152‐mediated cell death. These results suggest that PcCRN83_152 uses distinct mechanisms to achieve its virulence and cell death functions. CRN NCD variants were also shown to retain chromatin re‐localization capacities, pointing to a virulence function of PcCRN83_152‐mediated chromatin re‐localization.

Therefore, this work provides evidence suggesting that PcCRN83_152‐induced cell death is not required to enhance *P. capsici* virulence. This discovery will shed new light on our understanding of the virulence mechanisms used by CRN effector proteins.

## Results

### Random mutagenesis screen generates a library of PcCRN83_152 variants with an NCD phenotype

**Figure 1:**
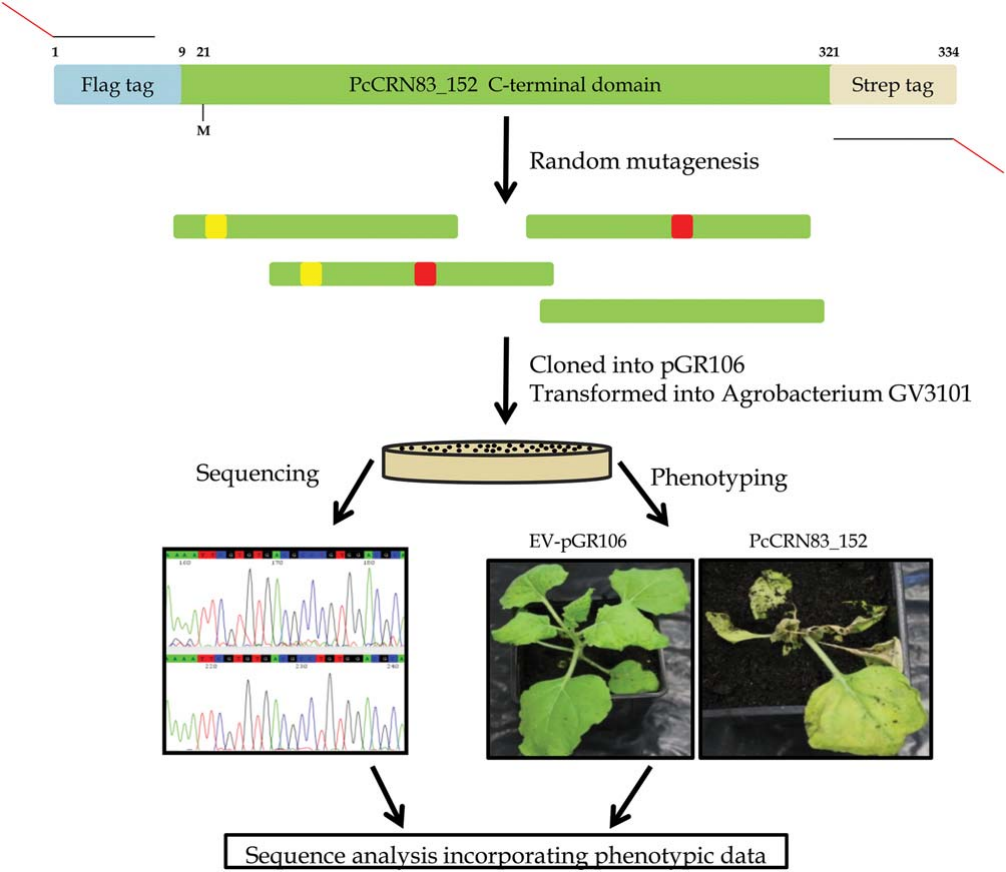
Random mutagenesis strategy. The PcCRN83_152 C‐terminal domain flanked with Flag and Strep tags was used for error‐prone polymerase chain reactions (PCRs). Primers were designed to contain In‐Fusion sites to allow direct cloning into pGR106 vector. The resulting PCRs were fused to pGR106 using In‐Fusion cloning and transformed directly into *Agrobacterium* strain GV3101. The resulting *Agrobacterium* colonies were screened by PCR for the presence of the desired insert and positive colonies were screened phenotypically by toothpick inoculation of *Nicotiana benthamiana* leaves. Simultaneously, inserts from these colonies were amplified with vector‐specific primers and sent for sequencing. The PcCRN83_152 C‐terminal domain first methionine (marked with an ‘M’) was used as the start of translation. Sequence and phenotypic analysis followed these procedures.

PcCRN83_152 over‐expression in *N. benthamiana* leaves induces plant cell death and promotes *P. capsici* virulence (Mafurah *et al*., 2015; Stam *et al*., 2013a, 2013b). To test whether these two distinct phenotypes are connected, a random mutagenesis approach was taken. Sequence corresponding to the PcCRN83_152 C‐terminal domain was amplified by error‐prone PCRs and cloned into a *Potato virus X*‐based vector (pGR106) (Jones *et al*., 1999), and subsequently sequenced and screened phenotypically for the presence of cell death (Fig. 1). Together with phenotypic assays, each putative effector variant was sequenced and analysed to generate a set of 506 candidate variants for which sequence data are available (Appendix S1, see Supporting Information). From this set, 307 variants were identified for which both high‐quality sequence and phenotypic information was available (for a summary of the sequence analyses, see Fig. S1 and Appendix S2 in Supporting Information).

First, we assessed the level of nucleotide mutations and consequent amino acid substitutions that were present in our variant library (Fig. S2, see Supporting Information). Our analyses showed that, on average, our library has 2.3 nucleotide mutations and 1.6 amino acid substitutions per variant. The library included 107 variants that lacked any amino acid substitutions. Importantly, all of these 107 variants showed a cell death phenotype, independently validating our screening approach and cell death phenotype (Fig. S2). The library consisted of 200 clones that encoded variants having one to seven amino acid substitutions. Importantly, and when assessing these variants on the basis of their phenotypes, a shift in the proportion of NCD‐inducing variants was evident when variants were classified into categories describing the number of amino acid substitutions per variant (Fig. S2). These results indicate successful combination of random mutagenesis and subsequent phenotypic screening in our approach.

Collectively, 62% of the amino acid residues located in the CRN83_152 C‐terminal region were substituted in at least one variant. We thus chose to assess which regions of PcCRN83_152 could be associated with cell death‐inducing activity. For this purpose, we plotted amino acid substitutions unique in either the NCD or cell death set against the PcCRN83_152 sequence targeted by mutagenesis (Fig. S3, see Supporting Information). Despite the presence of sequence regions in which specific substitutions were associated with loss of cell death, the screening depth and sequence coverage obtained in our approach prevented us from pinpointing specific regions or functions responsible for PcCRN83_152‐induced cell death.

### PcCRN83_152 NCD variants are stably expressed *in planta*

The PCR‐based random mutagenesis screen described above allowed the identification of PcCRN83_152 C‐terminal variants with a loss of cell death phenotype. To verify loss of cell death and exclude reduced stability as a cause of this phenotype, we sought to epitope‐tag, re‐clone and test these variants with conventional agroinfiltration assays. NCD variants with single amino acid changes were prioritized, together with variants for which some evidence of stability *in planta* was available (Table 1). This resulted in the selection of 14 NCD variants, which were cloned into the binary vector pB7WGF2 (Karimi *et al*., 2002), generating N‐terminal enhanced green fluorescent protein (EGFP) fusions suitable for detection and localization (Table 1).

**Table 1:** Summary of the PcCRN83_152 variants described in this study.

Variant	Amino acid changes	Cell death
2A10	L118I	+
2B5	L166M	–*
2B8	F63L	–
2F1	I130K	+
2F10	F160L	–
3H1	E27K; V131D	–
4A9	H140R	+
4B12	T21S; V82A	–
4C2	V150E	–
4D9	V100E	–
5E4	V97A	–
5H8	I66L; F160S; D170N; D259E	–
6D10	L191S	–
6E4	V89G	+

Presence or absence of cell death‐inducing capacity is indicated with a ‘+’ or ‘–’, respectively. Lack of stability *in planta* is indicated with *.

**Figure 2:**
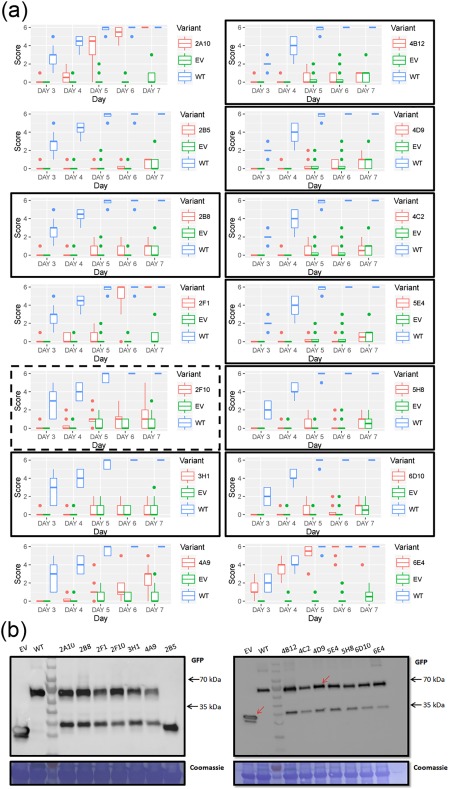
No cell death (NCD) variants show stability *in planta* and different rates of cell death‐inducing activity. (a) NCD variants, wild‐type PcCRN83_152 and green fluorescent protein (GFP) were over‐expressed in *Nicotiana benthamiana* leaves and cell death was scored using a scale of 0–6, where ‘0’ represents no cell death and ‘6’ represents completely dead plant tissue. Measurements were taken at 3, 4, 5, 6 and 7 days post‐infiltration. Variants with cell death levels not statistically different from the EV control (*P* < 0.05, Mann–Whitney *U*‐test) are surrounded with a black square. Variant 2F10 showed inconsistent levels of cell death induction and was not used for further experiments (dotted black line). (b) Representative leaf images were taken at 7 days post‐infiltration (for complete leaf images, see Fig. S4 in Supporting Information). Samples for Western blot were collected at 3 days post‐infiltration. Blots show that all the tested proteins, except 2B5, are expressed at levels similar to PcCRN83_152 wild‐type protein, and Coomassie blue staining of the gels is shown as a loading control. Red arrows indicate the bands with sizes corresponding to the PcCRN83_152 C‐terminal construct fused to GFP (≈ 61 kDa) and GFP alone (≈27 kDa). EV, empty vector; WT, wild‐type.

**Figure 3:**
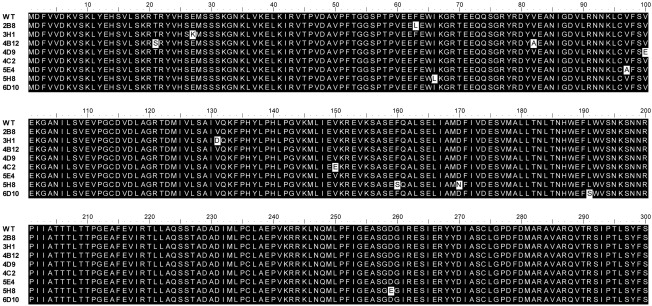
Protein alignment of PcCRN83_152 no cell death (NCD) variants. The amino acid sequences of the eight NCD variants described in this study were aligned with the amino acid sequence of PcCRN83_152 wild‐type (WT) protein. A black background indicates amino acids conserved with the wild‐type PcCRN83_152 protein, whereas a white background displays amino acids different from the wild‐type PcCRN83_152 amino acid sequence.

To assess the stability and verify the loss of cell death phenotypes, ectopic expression assays and Western blot analyses were conducted to measure and equalize expression levels to those found with the native variant (data not shown). Subsequent expression assays of these variants, together with the wild‐type, demonstrated slower cell death phenotypes for all variants (Fig. 2). Moreover, and consistent with our screening results, eight variants (2B8, 3H1, 4B12, 4C2, 4D9, 5E4, 5H8 and 6D10) consistently failed to induce cell death at any of the assessed time points, despite their accumulation inside plant cells (with the exception of 2B5, which was shown to be unstable *in planta*) (Figs 2 and S4, see Supporting Information). The expression of one variant (2F10) occasionally induced cell death, whereas the remaining mutants either showed reduced activity when compared with the wild‐type control (2A10, 2F1 and 4A9) or displayed similar levels of cell death induction when expressed *in planta* (6E4). The eight variants that showed a consistent and complete lack of cell death on over‐expression were selected for further analyses (Figs 2 and S4; Table 1). A protein alignment of these NCD variants is shown in Fig. 3.

### PcCRN83_152 NCD variants retain their ability to boost *P. capsici* infection

**Figure 4:**
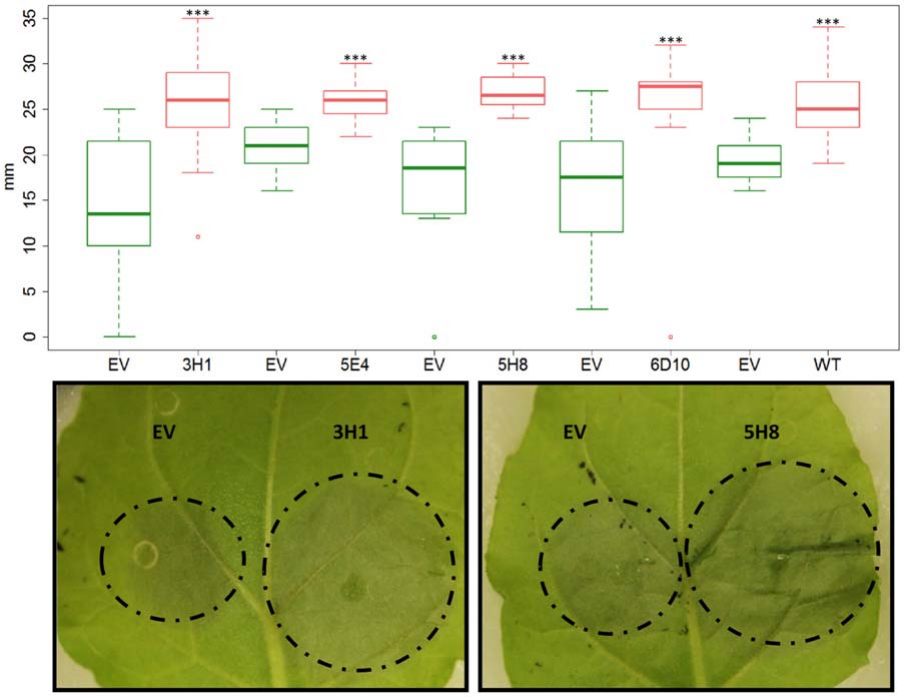
No cell death (NCD) variants boost *Phytophthora capsici* virulence. *Nicotiana benthamiana* leaves were infiltrated side by side with empty vector (EV) and PcCRN83_152 wild‐type (WT) or NCD variants. After 2 days, leaves were inoculated with *P. capsici* strain LT1534. The graph shows data from one of four independent experiments (full datasets are presented in Appendix S4, see Supporting Information). NCD variants that significantly affected *P. capsici* growth in at least three of four independent experiments are shown. NCD variants with inconsistent boost results were excluded from the graph. Lesion diameters were measured 3 days after infection and are shown in millimetres (mm). In these assays, a minimum of 15 infection sites were measured for each construct. Photographs show representative leaves for two of the NCD variants (3H1 and 5H8) at 3 days after infection. ***Significant difference as determined in a Mann–Whitney *U*‐test (*P* < 0.001).

PcCRN83_152 has been shown to boost *P. capsici* virulence when over‐expressed in *N. benthamiana* leaves (Mafurah *et al*., 2015; Stam *et al*., 2013a, 2013b). This has raised the possibility that cell death induced by PcCRN83_152 and the enhanced virulence observed previously reflect a causal relationship. To assess whether this is the case, we investigated whether NCD variants retained their ability to promote virulence. To test this, infection assays were performed in which *N. benthamiana* leaves were infiltrated to over‐express each variant alongside the wild‐type effector domain and an empty vector (EV) control. Subsequent infection of leaf panels and measurement of lesion growth revealed that four of the eight NCD variants tested retained their virulence activity, evidenced by their ability to consistently boost *P. capsici* infection (Fig. 4). These results suggest that PcCRN83_152 cell death is not required for virulence function.

### PcCRN83_152 NCD variants retain chromatin re‐localization capabilities

**Figure 5:**
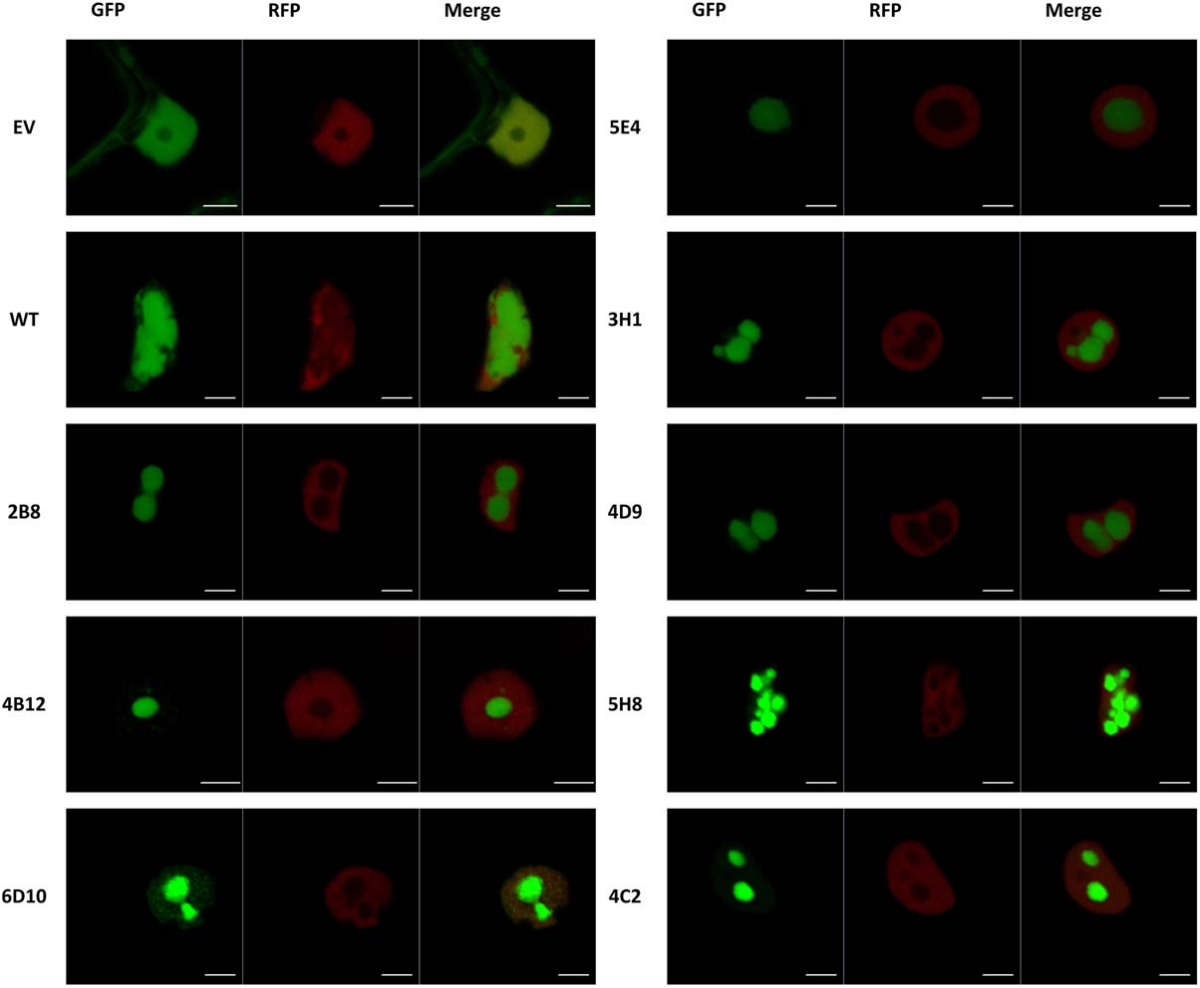
No cell death (NCD) variants retain chromatin re‐localizing capacity. Wild‐type PcCRN83_152 C‐terminal domain (WT), NCD variants tagged with green fluorescent protein (GFP) and GFP alone (EV) were expressed in transgenic mRFP‐H2B *Nicotiana benthamiana* plants. Confocal microscopy was performed at 2 days post‐infiltration. In contrast with EV‐GFP, PcCRN83_152 NCD variants were shown to accumulate in subnuclear bodies varying in shape and number. Nevertheless, these variants retained the capacity to re‐localize host chromatin, characteristic of PcCRN83_152 wild‐type protein. RFP, red fluorescent protein. Scale bar, 5 µm.

Previously, we have shown that ectopic expression of EGFP‐PcCRN83_152 in *N. benthamiana* epidermal cells results in a distinct subnuclear localization pattern that features patchy EGFP distribution in the nucleus (Stam *et al*., 2013a, 2013b). Interestingly, and in contrast with other cell death‐inducing CRN proteins, expression of EGFP‐PcCRN83_152 in *N. benthamiana* leaves leads to aberrant localization of host chromatin (Stam *et al*., 2013a), evidenced by the appearance of dark patches that are devoid of histone‐red fluorescent protein (RFP) signal and do not accumulate the nucleolar marker fibrillarin in co‐localization studies (Stam *et al*., 2013a). These observations have led to the suggestion that cell death is caused by CRN‐induced disruption of chromatin integrity (Stam *et al*., 2013a). To assess whether PcCRN83_152‐induced cell death is a result of chromatin re‐localization, the NCD variants were over‐expressed in leaves of *N. benthamiana* transgenic mRFP‐H2B plants and imaged by confocal microscopy (Fig. 5). All PcCRN83_152 NCD variants localized in the plant nucleus, demonstrating that it is not nuclear exclusion that leads to the observed NCD phenotypes. Furthermore, all the tested variants conserved their capacity to re‐localize host chromatin, evidenced by the emergence of multiple dark patches in histone‐RFP‐expressing nuclei (Fig. 5). Individual NCD variants, whilst retaining chromatin re‐localization features, showed a variety of subnuclear distributions. Therefore, Fig. 5 displays examples of the observed subnuclear localizations of individual NCD variants without implying that different NCD variants possess distinct subnuclear localization patterns. These results suggest that PcCRN83_152‐mediated chromatin re‐localization is not a consequence of the cell death events mediated by this CRN effector. In addition, it suggests that chromatin re‐localization, mediated by our NCD variants, reflects a virulence function.

### PcCRN83_152 NCD variants suppress PcCRN83_152‐mediated cell death

**Figure 6:**
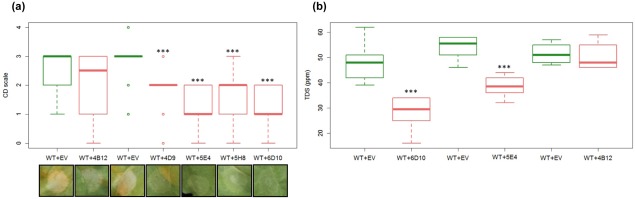
No cell death (NCD) variants suppress the PcCRN83_152 cell death phenotype. (a) PcCRN83_152 NCD variants or EV‐GFP were co‐expressed with the wild‐type version of the PcCRN83_152 C‐terminal domain (WT). Four NCD variants showed a suppressive effect on PcCRN83_152‐mediated cell death over at least two of three independent experiments (Appendix S4, see Supporting Information). For each experiment, at least 16 infiltration sites on 16 independent leaves were measured per construct. Cell death levels were assessed at 4 days post‐infiltration using a cell death scale of 0–6, where ‘0’ represents no cell death and ‘6’ indicates completely dead plant tissue. Variants with inconsistent suppression results were excluded from the graph presented here. Images of leaves displaying representative phenotypes, observed at 4 days after infiltration, are presented in Fig. S5 (see Supporting Information). (b) For three of these NCD variants, two that showed a suppression capacity (6D10 and 5E4) and one that did not (4B12), phenotypic assessment was complemented with ion leakage assays to confirm our phenotypic data. ***Significant difference in a Mann–Whitney *U*‐test (*P* < 0.001). CD, cell death; EV, empty vector; GFP, green fluorescent protein; TDS, total dissolved solids.

A kinase‐inactive variant of the *P. infestans* effector PiCRN8 and the *P. sojae* effector PsCRN115 have been shown to suppress cell death induced by CRNs highly similar to them (kinase‐active PiCRN8 and PsCRN63, respectively) (van Damme *et al*., 2012; Zhang *et al*., 2015a). To test whether PcCRN83_152 NCD variants suppress PcCRN83_152‐mediated cell death, PcCRN83_152 was co‐expressed with individual NCD variants. PcCRN83_152 cell death was consistently diminished when co‐expressed with four NCD variants (Figs 6a and S5, see Supporting Information). We selected two validated NCD variants and confirmed these results independently using ion leakage measurements (Fig. 6b). Interestingly, three NCD variants that consistently showed a virulence boost were also able to suppress PcCRN83_152‐induced cell death in co‐expression assays, providing further support for the idea that PcCRN83_152 cell death‐inducing activity does not represent a virulence function. Given that there is no strict correlation between cell death suppression and enhanced virulence phenotypes for all NCD variants, and that PcCRN83_152 also enhances virulence, it is unlikely that the suppression of CRN‐induced cell death impacts virulence in our assays.

**Figure 7:**
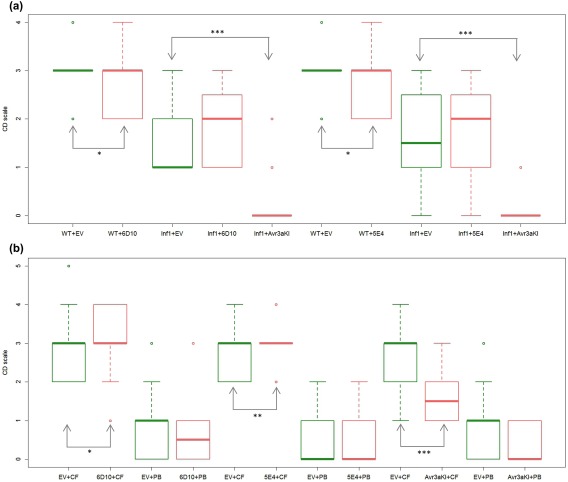
No cell death (NCD) variants do not suppress INF1‐ and culture filtrate (CF)‐mediated cell death. (a) PcCRN83_152 NCD variants, EV‐GFP and Avr3aKI were co‐expressed with the wild‐type version of PcCRN83_152 C‐terminal domain (WT) and INF1. Cell death levels were assessed at 4 days post‐infiltration using a cell death scale of 0–6, where ‘0’ represents no cell death and ‘6’ represents completely dead plant tissue. In this assay, 40 infiltration spots from 40 independent leaves were measured per construct (Appendix S4, see Supporting Information). (b) *Nicotiana benthamiana* leaves expressing both EV‐GFP and either PcCRN83_152 NCD variants or Avr3aKI (each on one side of the leaf) were infiltrated with either *Phytophthora capsici* CF or pea broth (PB). Cell death was scored 3 days after CF and PB infiltration. In this assay, 40 infiltration spots from 40 independent leaves were measured per construct (Appendix S4). ***Significant difference (*P* < 0.001, Mann–Whitney *U*‐test). **Significant difference (*P* < 0.01, Mann–Whitney *U*‐test) *Significant difference (*P* < 0.05, Mann–Whitney *U*‐test). CD, cell death; EV, empty vector; GFP, green fluorescent protein; WT, wild‐type.

CRN effectors have been shown to mediate the suppression of various cell death processes *in planta* (Rajput *et al*., 2014; Shen *et al*., 2013). In order to test whether the PcCRN83_152 NCD variants could work as general cell death suppressors, we co‐expressed two of these variants with *P. capsici* culture filtrate (CF) and with the *P. infestans* PAMP INF1 (Kamoun *et al*., 1997). The *P. infestans* effector Avr3aKI (Bos *et al*., 2006) was used as a positive control and, as expected, was capable of inhibiting CF‐ and INF1‐mediated cell death (Fig. 7). However, the two tested NCD variants failed to suppress both CF‐ and INF1‐mediated cell death (in contrast, 5E4 and 6D10 enhanced CF‐mediated cell death in our assays) (Fig. 7).

## Discussion

With the ever‐increasing availability of genome sequences for plant microbes, a large repertoire of effectors have been identified. However, despite valuable efforts, the deciphering of the functions of these numerous effector proteins still remains to be addressed. In relation to *Phytophthora* cytoplasmic effectors, an increasing number of studies have reported the elucidation of RXLR virulence functions (Anderson *et al*., 2015). CRN effectors, however, have received considerably less attention (Amaro *et al*., 2017). Recently, some studies have provided new insights into CRN virulence functions (Ramirez‐Garcés *et al*., 2015; Song *et al*., 2015; Zhang *et al*., 2015b), but the role of CRN‐mediated cell death has remained mostly elusive.

In this study, we made use of PcCRN83_152 features to address the role of cell death in virulence. PcCRN83_152 has a strong cell death phenotype and simultaneously enhances *P. capsici* growth (Mafurah *et al*., 2015; Stam *et al*., 2013b). By means of a PCR‐based random mutagenesis screen, we generated a library of 307 PcCRN83_152 variants, 108 of which had lost their cell death‐inducing capacities. Despite a high percentage of variants that had lost the ability to induce cell death, our analysis did not succeed in pinpointing the amino acid(s) or regions responsible for PcCRN83_152‐induced cell death. Our library contained amino acid substitutions for 62% of PcCRN83_152 C‐terminal amino acids. A higher coverage would be necessary to make definite conclusions about the specific regions connected with cell death‐inducing activities. Moreover, it is important to note that we did not test for protein stability in our screen, and so the regions required for PcCRN83_152 stability will be picked up in our analysis even if they are not directly connected with the PcCRN83_152 cell death phenotype.

Nonetheless, this screen generated PcCRN83_152 NCD variants that could be screened for virulence activities. From our library, 14 NCD variants were selected, eight of which did not show any cell death‐inducing activity even when expressed at similar levels as the PcCRN83_152 wild‐type protein (Fig. 2). It is important to note that all the selected NCD variants were more unstable when compared with the wild‐type protein, leading us to hypothesize that PcCRN83_152 may have important structural features that are affected in these variants.

These eight NCD variants with abolished cell death phenotype were tested for their capacity to enhance *P. capsici* virulence. Four of the eight variants were able to consistently boost *P. capsici* growth (Fig. 4), suggesting that cell death is not required for PcCRN83_152 virulence functions. However, we cannot exclude that cell death, despite not being required, aids PcCRN83_152 virulence function, as it is difficult to compare the levels of virulence boost between cell death‐ and non‐cell death‐inducing proteins when using different concentrations of *Agrobacterium* inoculum. Another hypothesis we cannot exclude is that the tested NCD variants may gain a new virulence function unrelated to that present in the PcCRN83_152 wild‐type protein. Although this appears to be quite improbable, the identification of PcCRN83_152 host targets and modification events should help to test this hypothesis. The observation that NCD variants retained nuclear localization and the capacity to re‐localize plant chromatin (Fig. 5) indicates that virulence function may have remained unaltered.

PcCRN83_152 NCD variants that are capable of enhancing *P. capsici* growth retain chromatin re‐localization capacities, suggesting a link between chromatin re‐localization and PcCRN83_152 virulence functions. However, although all tested NCD variants retained the capacity for chromatin re‐localization, only four were shown to consistently boost *P. capsici* virulence. Thus, it appears that chromatin re‐localization capacities are not sufficient *per se* for PcCRN83_152 virulence functions. Recently, two CRN effectors have been shown to directly bind DNA to achieve their virulence functions (Ramirez‐Garcés *et al*., 2015; Song *et al*., 2015). In addition, the targeting of DNA‐related processes has been predicted to be a conserved feature of CRN effectors (Zhang *et al*., 2016), making further investigation into the mechanisms involved in PcCRN83_152‐mediated chromatin re‐localization of upmost importance.

Despite their identification as cell death inducers (Torto *et al*., 2003), the induction of cell death is not a feature common to all CRN effectors (Haas *et al*., 2009; Shen *et al*., 2013; Stam *et al*., 2013b). On the contrary, several CRNs have been shown to work as general cell death suppressors (Shen *et al*., 2013). Even CRN effectors with high sequence similarity show opposite cell death phenotypes (Shen *et al*., 2013; Zhang *et al*., 2015a). Furthermore, a cell death and kinase‐inactive variant of the *P. infestans* effector PiCRN8 has been shown to suppress the cell death induced by the wild‐type form of PiCRN8 (van Damme *et al*., 2012). In this work, we showed that four of the tested PcCRN83_152 NCD variants consistently suppressed PcCRN83_152‐mediated cell death (Fig. 6). When testing two of these NCD variants for their ability to suppress cell death mediated by INF1 (Fig. 7a) and *P. capsici* CF, no suppressive activity was demonstrated (Fig. 7b). These results point to a possible dominant‐negative effect of the NCD variants on PcCRN83_152‐mediated cell death. As CRN effectors, including PcCRN83_152, have been suggested to form both homo‐ and heterodimers in plant cells (van Damme *et al*., 2012; Li *et al*., 2016), it is possible that cell death suppression by PcCRN83_152 NCD variants may occur through the modification of CRN complexes that are responsible for cell death.

Recently, cell death induced by PhCRN37 from *Plasmopara halstedii* has been suggested to be mediated by recognition in a sunflower resistant line (Gascuel *et al*., 2016). Thus, formally, it is possible that PcCRN83_152‐mediated cell death is caused by the recognition and activation of immunity and that our NCD variants avoid perception. Following this model, the PcCRN83_152 virulence boost would be retained despite evasion of recognition. If governed by effector‐triggered immunity (ETI), other *P. capsici* encoded effectors may act to suppress PcCRN83_152‐induced cell death *in vivo*. If this recognition hypothesis is true, the NCD variants could be suppressing PcCRN83_152‐mediated cell death by interfering with ETI‐associated processes activated by PcCRN83_152 recognition. Nevertheless, regardless of the mechanisms implied in this suppression, the fact that four NCD variants which were shown to boost *P. capsici* infection were also shown to suppress PcCRN83_152‐mediated cell death further points to a disconnection between PcCRN83_152‐induced cell death and virulence functions.

In summary, in this work, we addressed a question that has been overlooked in the CRN effector field, namely the virulence importance of CRN‐mediated cell death. A PCR‐based random mutagenesis screen enabled the identification of PcCRN83_152 variants which, despite complete absence of cell death‐inducing activity, retained the capacity to boost *P. capsici* virulence. Thus, the results of this study point to a separation of PcCRN83_152 cell death and virulence functions. Although it is not clear whether these findings hold true for other cell death‐inducing CRNs, this study provides new insights into CRN‐mediated cell death that need to be taken into account when trying to understand CRN virulence functions.

## Experimental Procedures

### Plant growth conditions

*Nicotiana benthamiana* plants were grown in a glasshouse under 16 h of light and a temperature of approximately 25/22 °C (day/night). The plants were kept in these conditions during all the experiments, unless stated otherwise.

### PCR random mutagenesis screen

PcCRN83_152 C‐terminal domain was PCR amplified using the primers 83_flag_F (5′‐GACTACAAAGACGATGACGACAAGGAGGGGGTAGTTGGCTCA‐3′) and 83_Phus_R (5′‐GGCGGTCGACGCGGCCGCTCACTTCTCGAACTGCGGGT‐3′). The resulting PCR band was gel purified using a MinElute Gel Extraction Kit (Qiagen, Hilden, Germany) and used as a substrate for another round of PCR amplification using the primers 83_Fus2_F (5′‐CACCAGCTAGCATCGATGACTACAAAGACGATGACGACAA‐3′) and 83_Fus2_R (5′‐GCCGCTCCAGGCGCGCCTCACTTCTCGAACTGCGG‐3′). This amplicon was subsequently cloned into the viral vector pGR106 (Jones *et al*., 1999) using the In‐Fusion HD cloning kit (Clontech, Mountain View, CA, USA). This construct was then used as a substrate to create a library of mutated PcCRN83_152 variants using the Diversify PCR Random Mutagenesis Kit (Clontech) according to the manufacturer's instructions. Two independent PCRs were performed, aiming to obtain an average of 2 and 3.5 nucleotide mutations per 1000 base pairs and using the primers 83_Fus2_F and 83_Fus2_R (sequences above). The primers used do not add a start codon to the PcCRN83_152 C‐terminal sequence. Translation is initiated at the methionine at position 12 of the PcCRN83_152 C‐terminal domain. The mutagenized amplicons were transformed into pGR106 using the In‐Fusion HD cloning kit (Clontech), and transformed into Stellar *Escherichia coli* cells (Clontech). Transformed cells were grown overnight in a 37 °C shaking incubator and plasmids were extracted using the Quiaprep Miniprep kit (Qiagen). These plasmid mixes were then transformed into *Agrobacterium tumefaciens* strain GV3101. Colonies resulting from these transformations were plated and PCR screened using vector‐specific primers: PVX2_F (5′‐CAAACTAGATGCAGAAACCATAAG‐3′) and PVX2_R (5′‐TTGACCCTATGGGCTGTGT‐3′). Amplicons from these PCRs were sent for sequencing with the same primers (PVX2_F and PVX2_R). Positive colonies were then toothpick inoculated into the leaves of 3‐week‐old *N. benthamiana* plants as described in Torto *et al*. (2003). Cell death and viral symptoms were assessed between 7 and 10 days post‐inoculation. Cases in which viral symptoms were not observed were excluded from further analysis.

### Sequence analysis

Initial sequence analysis was performed using CodonCode aligner package version 4.2.3 (CodonCode Corporation, Centerville, MA, USA). Using this program, sequence ends were trimmed, maximizing the region with an estimated error rate below 0.05%. Subsequently, variants with either forward or reverse sequences with less than 500 bases and a Phred quality score below 20 (estimated error rate at 1%) were removed from the analysis. Forward and reverse sequences for each variant were aligned and consensus sequences were generated (Appendix S1). Subsequently, consensus nucleotide and translated amino acid sequences were aligned using MUSCLE (V3.8.31). This allowed the identification of nucleotide mutations and corresponding amino acid substitutions in our dataset using Python Scripts (Appendix S3, see Supporting Information). Sequences containing mutations leading to frameshifts and premature stop codons were removed from the analysis. Moreover, to be additionally stringent on the nucleotide mutations identified, sequences containing nucleotide mutations with a Phred quality score of less than 30 (estimated error rate at 0.1%) were removed from the analysis.

### Re‐cloning of NCD variants

Selected PcCRN83_152 variants were PCR amplified from GV3101 cells using primers 83_cterm_F (5′‐CACCGAGGGGGTAGTTGGCTCA‐3′) and 83_cterm_R (5′‐TCACTTCTCGAACTGCGG‐3′). They were then recombined into the entry vector pENTR/D‐TOPO using the pENTR Directional TOPO cloning kit (Thermo Fisher Scientific, Waltham, MA, USA) and sequence verified. Correct constructs were used for recombination into the binary vector pB7WGF2, with an N‐terminal GFP fusion and a 35S promoter element, using Gateway LR reactions (Invitrogen, Paisley, UK). Constructs were sequence verified and transformed into *A. tumefaciens* strain AGL1. Wild‐type PcCRN83_152, cloned and tested as in Stam *et al*. (2013b), was used in the same vector (pB7WGF2) and *Agrobacterium* strain (AGL1). Avr3aKI and INF1 were used in the pGRAB vector.

### CRN cell death assays

All constructs were prepared for infiltration as described in Stam *et al*. (2013b). For cell death assays, cultures were mixed with *A. tumefaciens* AGL1 cells carrying the silencing suppressor p19, achieving final optical densities (ODs) of 1.0 for CRN NCD variants, 1.0 for p19, 1.0 for EV, 1.0 for Avr3aKI, 0.25 for PcCRN83_152 wild‐type and 0.25 for INF1. PcCRN83_152 wild‐type was used at a lower OD to ensure similar levels of protein expression, as PcCRN83_152 variants showed less stability *in planta*. Scoring was performed between 2 and 7 days according to the experiment.

Cell death scoring was performed using a scale of 0–6 as described in Stam *et al*. (2013b). For ion leakage measurements, six leaf discs from infiltrated leaves were collected, placed in 10 mL of Milli‐Q water and shaken at 30 rpm at room temperature for 2 h. Total dissolved solids (TDS) were then measured in solution using a Primo pocket TDS tester (Hanna Instruments, Woonsocket, Rhode Island, USA). For each point and treatment, six measurements were taken. *Phytophthora capsici* CF and pea broth (PB) were produced as described in Stam *et al*. (2013a).

### Infection assays

*Phytophthora capsici* growth assays were performed on *N. benthamiana* leaves that had been infiltrated with appropriate *Agrobacterium* constructs using the ODs described above. Two days after infiltration, leaves were drop inoculated with 5 µL of zoospore solution (50 000 spores/mL) from *P. capsici* strain LT1534. Lesion diameters were measured at 3 days post‐inoculation.

### Western blotting

To test for the stability of PcCRN83_152 NCD variants, plant tissue, infiltrated with the respective constructs at the same conditions as used for the cell death assays, was harvested at 2 days post‐infiltration and frozen in liquid nitrogen. Protein extractions were performed as in Stam *et al*. (2013a). Protein extracts were run on Bio‐Rad (Bio‐Rad, San francisco, CA, USA) TGX gels before being transferred to poly(vinylidene difluoride) (PVDF) membranes using a Bio‐Rad Trans Blot Turbo Transfer System. Blots were blocked for 30 min with 5% milk in TBS‐T (0.1% Tween 20) and probed with GFP antibody (Santa Cruz, Dallas, Texas, USA) (1 : 2500), followed by anti‐mouse horseradish peroxidase (HRP) antibody (Santa Cruz) (1 : 20 000). Blots were incubated with SuperSignal West Femto Maximum Sensitivity Substrate (Thermo Fisher Scientific) and imaged on a Syngene (Cambridge, UK) GBox TX4 Imager. After imaging, for the visualization of total protein levels, membranes were Coomassie dyed using Imperial protein stain (Thermo Scientific, Waltham, MA, USA) according to the manufacturer's instructions.

### Confocal imaging

For confocal microscopy, constructs were infiltrated as described above and ODs were adjusted to a final OD of 0.05 for all constructs without the presence of p19. For these assays, transgenic *N. benthamiana* mRFP‐H2B plants were used. Confocal imaging was performed at 48 h post‐infiltration on a Zeiss (Jena, Germany) LSM 710 confocal microscope with a W Plan‐Apochromat 40X/1.0 DIC M27 water dipping lens and using the following settings: GFP (488 nm excitation and 400–600 nm emission) and monomeric RFP (mRFP) (561 nm excitation and 400–700 nm emission).

## Supporting information

Additional Supporting Information may be found in the online version of this article at the publisher's website:

**Fig. S1** Sequence analysis pipeline. Using CodonCode Aligner software base calling and end trimming capacities, consensus sequences were generated for 506 PcCR83_152 variants (Appendix S1). From these 506 variants, 66 contained gaps or inserts and were removed from the analysis. After this, another quality trimming step was performed in which all the sequences that contained nucleotide mutations in positions with Phred base calling quality of less than 30 were removed, leaving 371 sequences. The final 307 sequences were obtained by removing variants without conclusive phenotypic data, encoding premature stop codons or with amino acid substitutions in the start codon.

**Fig. S2** Characterization of PcCRN83_152 library of variants. (a) Number of clones with either cell death (CD) or no cell death (NCD) phenotype according to the number of nucleotide (nt) mutations they contain. (b) Number of sequences with either CD or NCD phenotype according to the number of amino acid (aa) substitutions they contain.

**Fig. S3** Distribution of amino acid substitutions across PcCRN83_152 C‐terminal sequence. The amino acid substitutions presented uniquely in the no cell death (NCD) or cell death (CD) set of the PcCRN83_152 library of variants were plotted against the wild‐type PcCRN83_152 amino acid sequence. Letters refer to amino acids and colours to amino acid characteristics according to the Lesk colour code (small non polar, orange; hydrophobic, green; polar, magenta; negatively charged, red; positively charged, blue) (Lesk, 2002). Single amino acid substitutions that were identified as leading to an NCD phenotype are displayed within a bold square.

**Fig. S4** Full images used for Fig. 2. *3C11 was removed from this study for presenting an incorrect mutational profile.

**Fig. S5** Full images used for Fig. 6. *3C11 was removed from this study for presenting an incorrect mutational profile.

**Appendix S1** Sequencing data for the 506 CRN83_152 variants analysed in this study.

**Appendix S2** Mutation profile of the 307 clones analysed in this study.

**Appendix S3** Python script used for sequence analyses.

**Appendix S4** Raw scoring data used for Figs 2, 4, 6 and 7.
