# Supplementary figures and images for: Random mutagenesis screen shows that Phytophthora capsici CRN83_152‐mediated cell death is not required for its virulence function(s)
Source: Mol Plant Pathol. 2017 Oct 24;19(5):1114–26. doi: 10.1111/mpp.12590 (PMC5947615; doi:10.1111/mpp.12590)

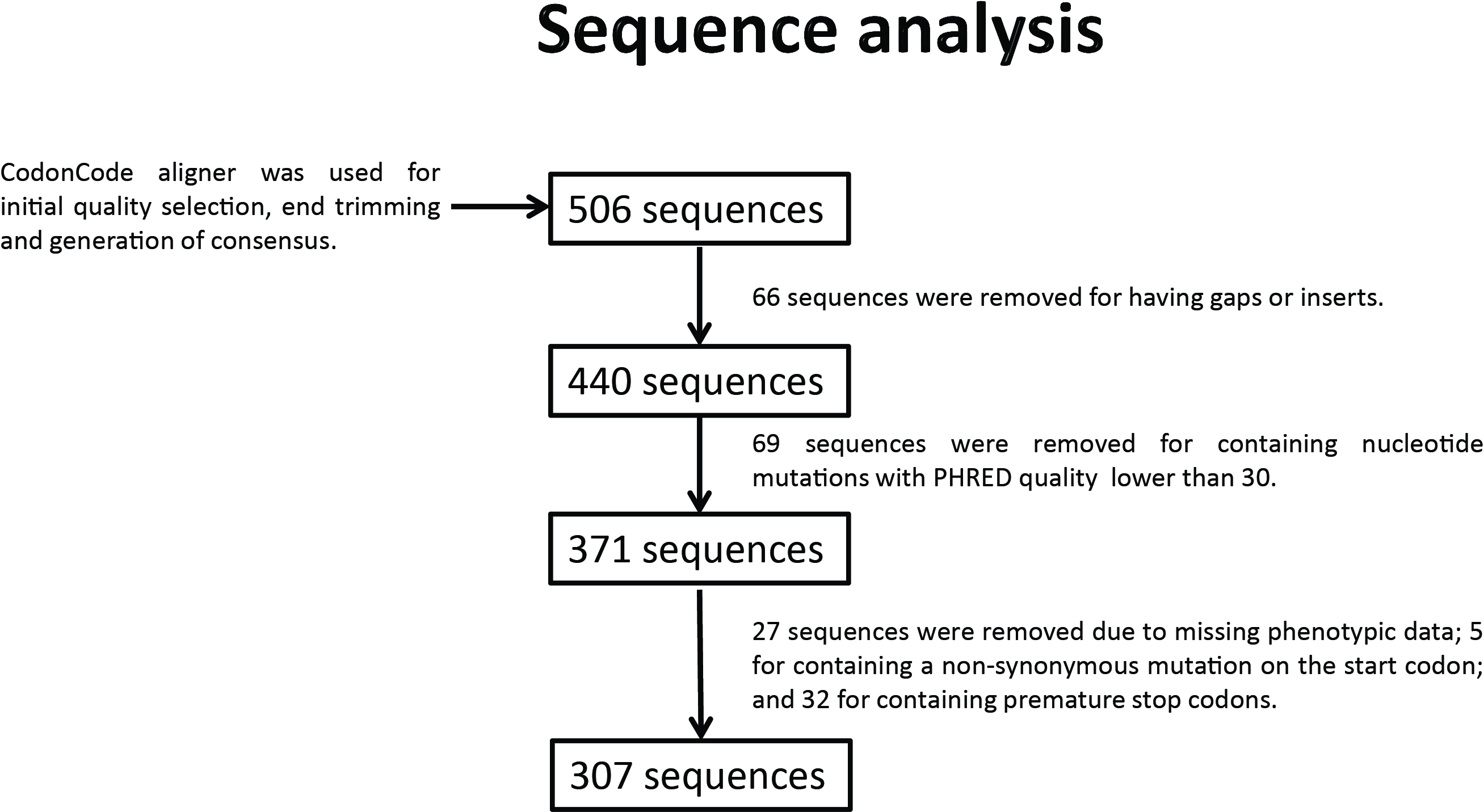

Supplement: Supplementary file 1 — Fig. S1 Sequence analysis pipeline. Using CodonCode Aligner software base calling and end trimming capacities, consensus sequences were generated for 506 PcCR83_152 variants (Appendix S1). From these 506 variants, 66 contained gaps or inserts and were removed from the analysis. After this, another quality trimming step was performed in which all the sequences that contained nucleotide mutations in positions with Phred base calling quality of less than 30 were removed, leaving 371 sequences. The final 307 sequences were obtained by removing variants without conclusive phenotypic data, encoding premature stop codons or with amino acid substitutions in the start codon. [file MPP-19-1114-s001.jpg]

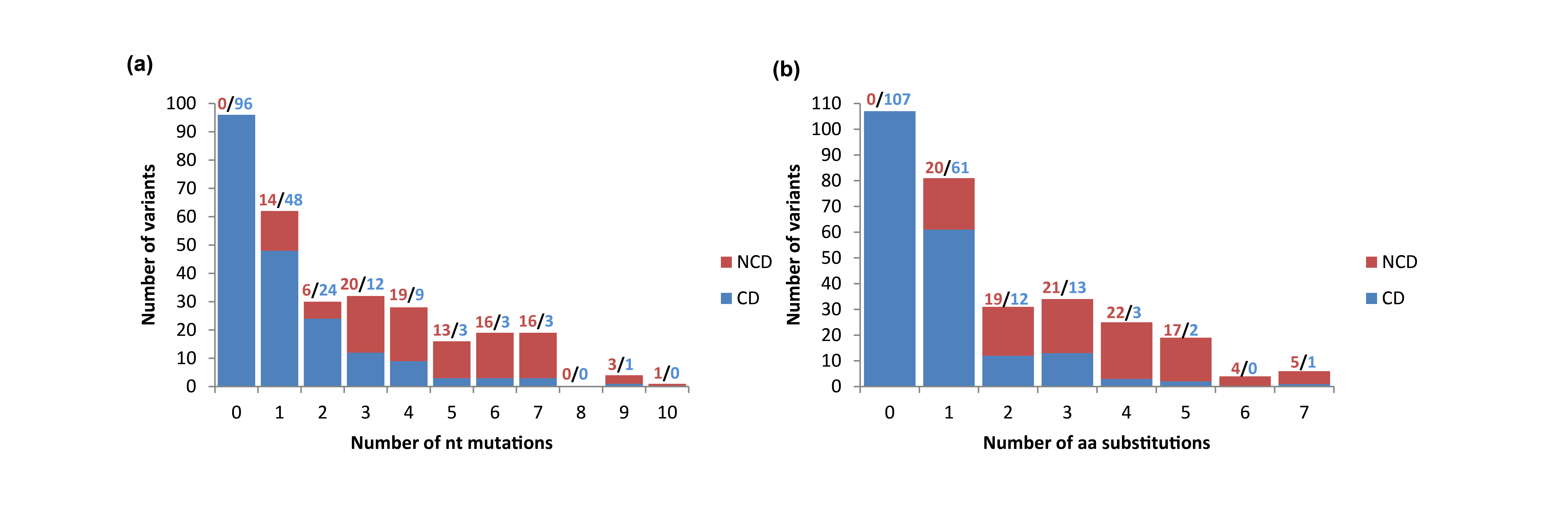

Supplement: Supplementary file 2 — Fig. S2 Characterization of PcCRN83_152 library of variants. (a) Number of clones with either cell death (CD) or no cell death (NCD) phenotype according to the number of nucleotide (nt) mutations they contain. (b) Number of sequences with either CD or NCD phenotype according to the number of amino acid (aa) substitutions they contain. [file MPP-19-1114-s002.jpg]

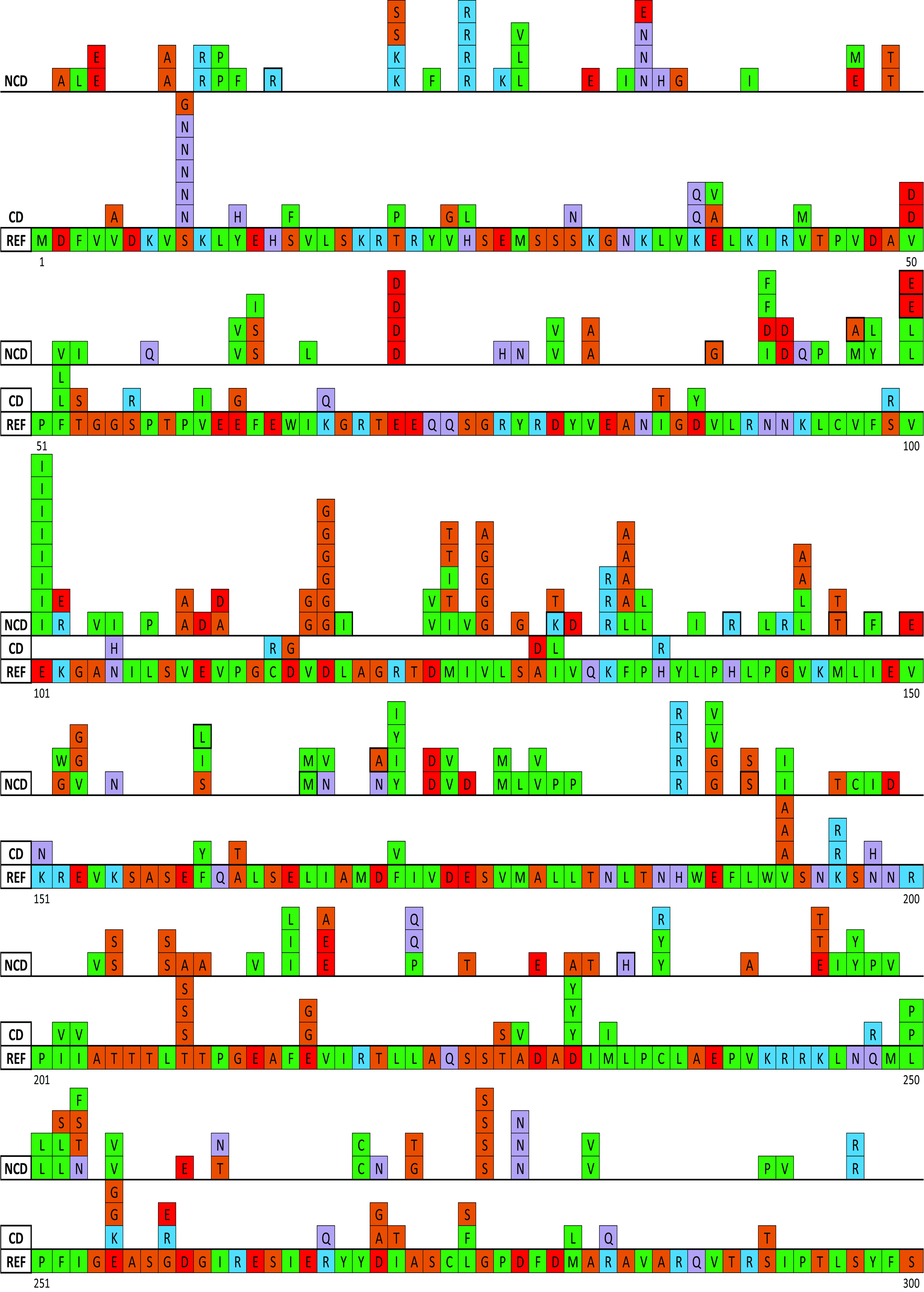

Supplement: Supplementary file 3 — Fig. S3 Distribution of amino acid substitutions across PcCRN83_152 C‐terminal sequence. The amino acid substitutions presented uniquely in the no cell death (NCD) or cell death (CD) set of the PcCRN83_152 library of variants were plotted against the wild‐type PcCRN83_152 amino acid sequence. Letters refer to amino acids and colours to amino acid characteristics according to the Lesk colour code (small non polar, orange; hydrophobic, green; polar, magenta; negatively charged, red; positively charged, blue) (Lesk, 2002). Single amino acid substitutions that were identified as leading to an NCD phenotype are displayed within a bold square. [file MPP-19-1114-s003.jpg]

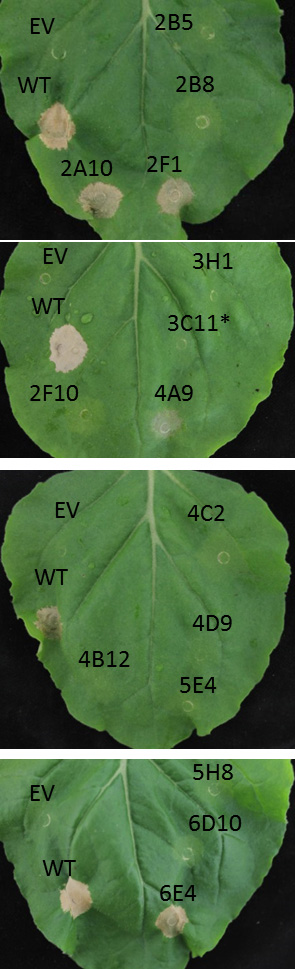

Supplement: Supplementary file 4 — Fig. S4 Full images used for Fig. 2. *3C11 was removed from this study for presenting an incorrect mutational profile. [file MPP-19-1114-s004.jpg]

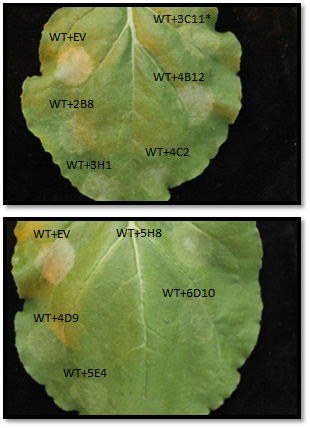

Supplement: Supplementary file 5 — Fig. S5 Full images used for Fig. 6. *3C11 was removed from this study for presenting an incorrect mutational profile. [file MPP-19-1114-s005.jpg]
